# Generalization of Clustering Coefficients to Signed Correlation Networks

**DOI:** 10.1371/journal.pone.0088669

**Published:** 2014-02-21

**Authors:** Giulio Costantini, Marco Perugini

**Affiliations:** Department of Psychology, University of Milan-Bicocca, Milan, Italy; Leibniz-Institute for Farm Animal Biology (FBN), Germany

## Abstract

The recent interest in network analysis applications in personality psychology and psychopathology has put forward new methodological challenges. Personality and psychopathology networks are typically based on correlation matrices and therefore include both positive and negative edge signs. However, some applications of network analysis disregard negative edges, such as computing clustering coefficients. In this contribution, we illustrate the importance of the distinction between positive and negative edges in networks based on correlation matrices. The clustering coefficient is generalized to signed correlation networks: three new indices are introduced that take edge signs into account, each derived from an existing and widely used formula. The performances of the new indices are illustrated and compared with the performances of the unsigned indices, both on a signed simulated network and on a signed network based on actual personality psychology data. The results show that the new indices are more resistant to sample variations in correlation networks and therefore have higher convergence compared with the unsigned indices both in simulated networks and with real data.

## Introduction

Network analysis is a family of powerful tools that constitute the basis of important insights in many fields of science (for introductions to network analysis, see [1]–[5]). A network is an abstract and flexible representation of several entities, represented as a set of nodes or vertices (*V*), and of their relationships, represented as a set of edges (*E*) that connect the nodes. Networks are used to represent various systems, such as friendships [6], scientific collaborations [7], the world-wide-web [8], the co-expression of genes [9], air transportation [10] and the brain [11]. Recently, network analysis has been applied to psychological phenomena such as personality and psychopathology [12]–[16].

A network is said to be unweighted or binary if any edge 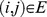 can be either absent or present, whereas if the intensity of the ties is coded, the network is said to be weighted. An unweighted network of size *n* can be represented by an 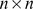 adjacency matrix (*A*) whose elements 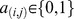 convey information about the presence or the absence of an edge. In this paper, we deal only with undirected networks; therefore, 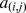 is assumed equal to 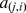. All of the diagonal elements 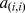 are assumed equal to zero. A weighted network can be represented by a matrix of weights (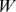) that associates a value 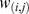 to the edges. For the sake of simplicity and without loss of generality, we consider 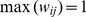. Weighted networks provide a more accurate representation of phenomena characterized by a non-negligible heterogeneity in the intensity of the connections. For instance, weighted networks allow for representing the importance of a stable scientific collaboration compared with an occasional coauthorship or the importance of a stable connection between two airports that can carry thousands of passengers a day compared with connections that are operated occasionally [17]. The necessity of more precise models has led researchers to increase their interest in weighted networks and therefore to include edge weights in the computation of key network statistics (e.g., [17]–[19]).

In the typical applications of network analysis, the weights represent the intensity or the capacity of a relationship and are therefore positive numbers (e.g., [1], [17]); however, a network can also include relationships that are naturally represented by considering both positive and negative edges. Examples are social networks in which both liking and disliking relationships are allowed [20]–[23]. A signed network can be represented by a signed adjacency matrix whose elements take value 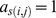 if *i* and *j* are connected by a positive edge, if *i* and *j* are connected by a negative edge, and 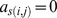 if no edge connects the nodes. A signed weighted network can be represented by a signed weights matrix 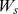 that associates to each edge a weight reflecting both the sign and the strength (i.e., absolute value) of the connection.

The meanings of nodes, ties and weights vary among different applications of network analysis: this contribution focuses especially on networks in which nodes are defined by variables and ties are defined by their correlations. This type of network has been used often in the field of weighted gene co-expression network analysis [9], and it is at the basis of the definition of networks in personality psychology and psychopathology [13], [14], [24]. The correlation coefficient naturally assumes both positive and negative values, but for some applications, edge signs are typically neglected: the clustering coefficient [19], [25] represents a primary example of such a strategy [9], [14], [24]. The clustering coefficient assesses the connectivity in a node’s neighborhood: a node has a high clustering coefficient if its neighbors tend to be directly connected with each other. The coefficient is fundamental to assessing the small-world property [26], and it can be interpreted as an index of the redundancy of a node [5], [27]–[29]. This last property is particularly important in personality and psychopathology networks. These networks are usually based on personality questionnaires: nodes represent questionnaire items and edges represent their correlations [13]. Given that questionnaire items can sometimes tap into similar issues, the identification of the most redundant nodes in a network could help in identifying items that do not add unique information to the network.

The aim of this article is to generalize clustering coefficients to signed correlation networks. The remainder of this paper is organized as follows. First, we formally present the clustering coefficient for both unweighted and positively weighted networks. Second, we discuss why a generalization to the signed case is needed. Third, we propose modifications of the indices to extend their use to signed correlation networks. Finally, we show the performance of the new indices using both simulated networks and networks based on real data.

### Definition of the Clustering Coefficient for Unweighted and Weighted Networks

A triangle is a subgraph of three nodes all connected to each other. It can be conceived of as a direct connection of a node *j* with a node *q*, given by *(j, q)*, plus an indirect connection that travels through another node, *i*, given by *(j, i, q)*. If the direct edge *(j, q)* is null, the indirect path that travels through *i* is especially important because it conveys unique information about the relationship between *j* and *q*. In this case, the missing direct edge between *j* and *q* is said to constitute a structural hole [28]. Conversely, if the direct edge *(j, q)* is present, the importance of the indirect path is reduced and *i* can be considered redundant in establishing a connection between *j* and *q*. This idea can be applied to the whole neighborhood of a node *i*; the local clustering coefficient was initially defined by Watts and Strogatz [25] for unweighted networks as the number of connections among the neighbors of a focal node over the maximum possible number of such connections,
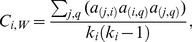
(1)where 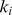 is the degree of node *i* [30]. The clustering coefficient can be equivalently conceived of as the number of triangles in the neighborhood of a focal node (*t_i_*), normalized by the maximum possible number of such triangles,



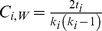
(2)and it can be interpreted as a measure of how much a focal node *i* is redundant in establishing connections in its neighborhood [5], [29].

Several generalizations of the clustering coefficient have been proposed for positively weighted networks [19]. We consider here two generalizations that are well-known, proposed by Onnela and colleagues [31] and from Zhang and Horvath [9], [32]. Onnela and colleagues defined the intensity of a subgraph in a network as the geometric average of its edge weights and proposed a weighted version of the clustering coefficient by substituting the number of triangles in the numerator of (1) with the sum of triangle intensities
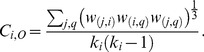
(3)


This index requires an underlying binary network for computing the unweighted degree in the denominator [32] and takes into account the weights of all edges in the triangles [19].

Zhang and Horvath [9] generalized the clustering coefficient to networks with positive weights,
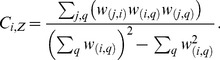
(4)


The numerator of (4) is a generalization of Watts and Strogatz’s clustering coefficient [25] to a matrix of weights instead of to the adjacency matrix, whereas the denominator represents the maximum possible value that can be obtained by the numerator, such that 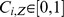. It can be equivalently expressed with the formula
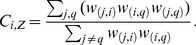
(5)


Both 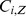 and 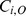 coincide with 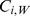 if binary edge weights {0, 1} are considered [19], [32]. In contrast to Onnela’s formulation, 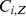 is not influenced by the weights of all the edges, being insensitive to the weights of the edges incident to *i* [19].

### Why is a Generalization of Clustering Coefficient to Signed Networks Needed?

In the framework of balance theory, the sign of a cycle is the product of the signs of its lines, and the degree of balance of the network has been defined as the proportion of positive cycles [20]. Starting from this framework, Kunegis and colleagues operationalized the concept of multiplicative transitivity for signed networks as the tendency for any two incident edges “to be completed by a third edge having as a weight the product of the two edges’ weights” [21]. Relying on the concept of multiplicative transitivity, they also showed that it is possible to predict the edge signs in a social network by using the signs in the square adjacency matrix 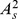, in which each entry is the sum of the signs of the length-2 paths between any pair of nodes *i* and *j*. If there are more positive than negative paths joining two nodes, then the predicted direct path between them is positive. Otherwise, the predicted direct path is negative. Consider the task of guessing whether two individuals, John and Paul, are friends or enemies by knowing their relations with other people. If they have many friends and/or many enemies in common, it is also likely that they are friends themselves, while if the friends of Paul are in general the enemies of John and vice versa, John and Paul are more likely to be foes. Similarly, the evolution of a social connection between two individuals can be modeled as a function of the product of the signed links among the two focal individuals and their common neighbors [23]. If Mary and Anne have many friends and/or enemies in common, it is likely that they will become friends themselves, while if the enemies of Mary are the friends of Anne and vice versa, it is likely that they will become foes.

The distinction between positive and negative triangles is relevant not only in social networks but also in correlation networks, especially for assessing the redundancy of a node. 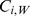, 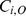 and 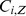 can all be interpreted as measures of redundancy [5], [29], but this interpretation is only meaningful as long as the presence of a direct path (*j*, *q*) makes the indirect path (*j*, *i*, *q*) less important or less informative. Conversely, when the sign of the direct path is different from the sign of the indirect path (computed as the product of the edge signs), if one attempted to predict the sign of the direct edge using just the indirect path [21], one would hypothesize a relationship of exactly the opposite sign between *j* and *q* relative to the one expressed by the direct edge. In this case, the information conveyed by the indirect path cannot be considered redundant with regard to that of the direct path. In the case of correlation networks, in which nodes represent variables and edge weights their connections, note that simply reversing one or more variables cannot convert a negative triangle into a positive one. Reversing a variable changes the signs of two of the connections of the triangle, but being the sign of a triangle defined by the product of the signs of its edges, this modification leaves the sign of the triangle unchanged. Reversing a variable can change the sign of the direct edge and of the indirect path together, but not the sign of one of the two independently of the other.

When 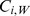, 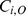 and 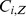 are applied to a signed network considering the absolute values of weights, they do not distinguish negative from positive triangles and cannot be interpreted as indices of redundancy for those nodes that are involved in negative triangles. Therefore, in this work, we propose adaptations of 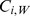, and 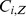 such that positive triangles are considered positively and negative triangles are considered negatively in the summation. The signed clustering coefficient of a node *i* is high (low) if the pairs of nodes that have a connection of the same sign to *i* are also connected by a positive (negative) edge and if the pairs of nodes that have a connections of opposite signs with *i* are more likely to be connected by a negative (positive) edge. The signed clustering coefficient is high if the node *i* is generally involved in triangles with 0 or 2 negative edges and is low if *i* is generally involved in triangles with 1 or 3 negative edges.

An important reason to consider signed versions of 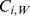, and 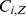 is that the signed indices are expected to be more resistant than the corresponding unsigned indices to the presence of noise. Correlation networks are typically based on sample estimation: especially when the sample size is not large, many small correlations might still be unstable estimates of the population values [33]. These small correlations tend to form a large number of very small triangles that are expected to be equally distributed among positive and negative: although in the computation of the unsigned indices they can have a large influence, their effect should cancel out when computing the signed indices given that the negative triangles are subtracted and the positive triangles are added in the computation of the indices.

### The New Signed Indices of the Clustering Coefficient

The unweighted clustering coefficient can be generalized to signed networks by simply replacing the unsigned adjacency values with the signed values in the formula
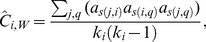
(6)where the degree (*k_i_*) in the denominator is computed considering the unsigned values. The index 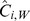 varies in [−1,1] and assumes the values 1 and -1 when all of the *i*’s neighbors are directly connected in pairs and these pairs form only, respectively, positive and negative triangles with *i*. The value zero indicates that *i* participates in positive and negative triangles in equal number or that no edge connects *i’*s neighbors to each other.

Additionally, 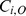 can be similarly generalized to signed networks by replacing the unsigned weights with the signed ones in Formula (3): in Formula (7), when the sign of a triangle is negative, the intensity of that triangle is subtracted in the sum
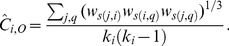
(7)



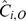
 varies in [−1,1] and takes value 1 if all of *i*’s pairs of neighbors form only positive triangles with *i* and the weights of all such connections are equal to one in absolute value; it takes value −1 if all of *i*’s pairs of neighbors form only negative triangles with *i* and the weights of all such connections are equal to one in absolute value, and it takes value 0 if the positive and negative triangles in which *i* participates are balanced or if the neighbors of *i* are all disconnected from each other. In correlation networks, exactly null correlations are unlikely: if one considers all the non-null correlations in the computation of the degree in (3) and (7), the denominator becomes a constant that is dependent solely on the size of the network. The alternative possibility would be to set the correlations that are below a threshold to zero; however, this procedure has important theoretical disadvantages [9]. Moreover, although small correlations can be individually unreliable estimates of the population values, they can convey reliable information when they are considered together (e.g., [34]), and their exclusion from the computation would ultimately result in loss of information. Therefore, we suggest considering all of the edges in the computation of both the numerator and the denominator.

The generalization of 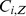 is slightly less straightforward. In its original formulation, 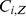 includes the weights of the indirect paths both in the numerator and in the denominator, making the index particularly sensitive to the direct paths *(j, q)* of the triangles, which is included in the numerator but not in the denominator (cf. Formula 5). If the unsigned weights were replaced with the signed weights both in the numerator and in the denominator, the index would be dependent especially on the sign of the direct paths in the neighborhood of *i*. Making the index sensitive to the sign of the direct path would be particularly problematic in correlation networks, in which nodes represent variables and reversing a variable can arbitrarily change the signs of the direct path and of the indirect path, even if it cannot change the sign of the triangle. This would make the sign of the clustering coefficient dependent on the variable orientation. For instance, recoding a variable from “extraversion” to “introversion” and changing the signs of the correlations consequently would change the clustering coefficients. Therefore, we propose a generalization in which the numerator considers the signed weights and the denominator considers the weights in absolute value:
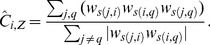
(8)



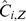
 varies in [−1,1] and takes value 1 if all of *i’*s pairs of neighbors form only positive triangles with *i* and the absolute weights of the edges between the neighbors are equal to 1 (irrespective of the absolute weights of the indirect paths); it takes value −1 if all of *i’*s neighbors form only negative triangles with *i* and all of the absolute weights of the direct edges between *i*’s neighbors are equal to 1, and it takes value 0 if the positive and negative triangles in which *i* participates are balanced or if *i*’s neighbors are disconnected from each other.

Kunegis and colleagues [21] introduced a measure of global clustering coefficient for signed networks, 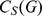. 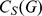 and 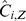 differ in the fact that whereas the first is a property of the network (global clustering coefficient, e.g., [53]), the second is a property of each node in the network (local clustering coefficient). Some similarities between 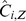 and 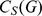 become apparent if we express 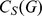 as
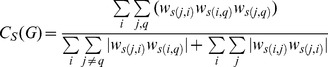
(9)and compare it with Formula (8). The numerator of 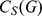 is equal to the sum of the numerators of 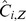 for all of the nodes, whereas the denominator of 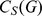 is equal to the sum of the denominators of 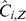 of all of the nodes plus the term 
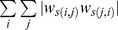
. In undirected networks, in which 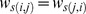, this last term is equal to the sum of all of the squared elements of the weight matrix.

**Figure 1:**
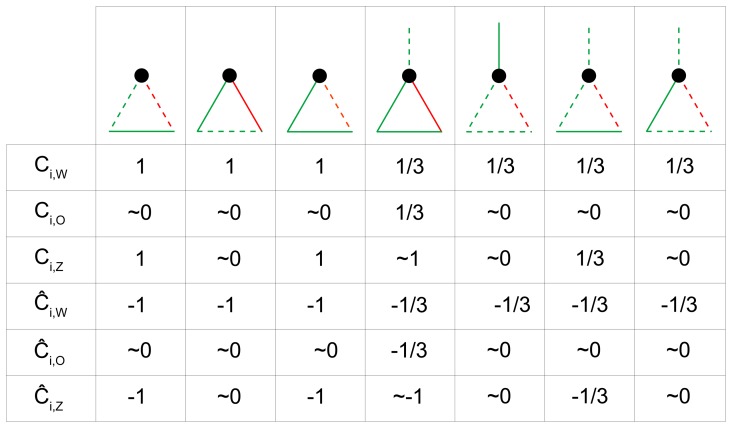
Examples of clustering coefficients for different sign and weight configurations. 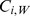
, 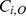 and 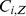 are the clustering coefficient indices proposed by [25], [31] and [9], respectively. 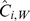, 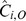 and 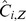 are the corresponding indices generalized to the signed case. Solid lines (–) represent edges of weight equal to 1 in absolute value, and dashed lines (–) represent edges of weight close to 0. Green lines are positive and red lines are negative. Edge weights are ignored in the computation of the unweighted clustering coefficients 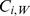 and 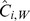. In each triangle one edge is negative. Note however that it is irrelevant for the value of the signed clustering coefficients which of the three edges is the negative one. We considered the case of a negative triangle with one negative edge, but we could have equally considered the case of a negative triangle with three negative edges without affecting the results.

Figure 1 shows the values of the unsigned and the signed indices for the case in which the focal node participates in a single negative triangle. The examples are those shown in Saramäki et al. (2007, fig. 1), with the main difference that one edge in each triangle is negative. To illustrate the properties of the proposed indices of the clustering coefficient, we tested them on simulated networks and on networks based on real data. Based on the definitional features of these indices, we hypothesize that:

1. The signed and the unsigned indices of the clustering coefficient should have an increasingly strong correlation as a function of the presence of positive triangles in the network, and they should diverge as a function of the presence of negative triangles.
2. The signed indices of the clustering coefficient should be consistently more resistant to the presence of noise in correlation networks, compared with the unsigned indices, and should therefore show higher agreement.

The analyses were performed with R using packages *qgraph* [35], *WGCNA* [36], [37], *Matrix* [38] and *psych* [39]. Functions for computing the new indices of clustering coefficient have been included in package *qgraph*.

## Study 1: Simulated Networks

The aim of this simulation was to inspect how the unsigned and signed formulations of the clustering coefficient converge in correlation networks as a function of the proportion of negative triangles in the network. Furthermore, we manipulated the presence of noise in the network to test how the indices were affected by the presence of completely random correlations.

### Method

**Figure 2:**
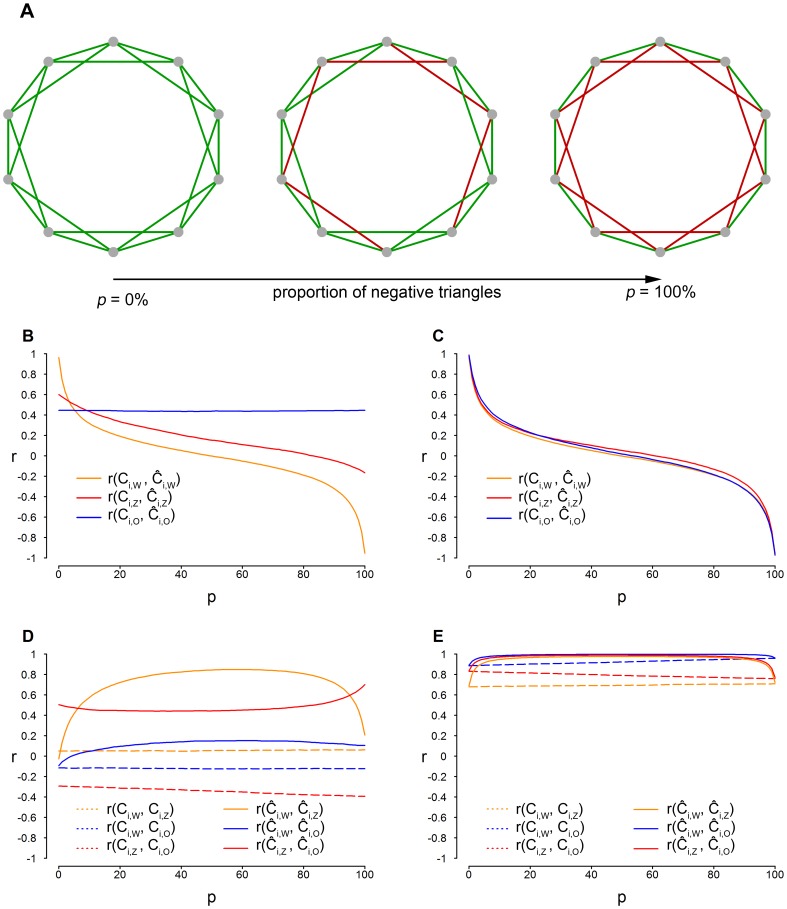
Correlation (*r*) between clustering indices as a function of the proportion of negative triangles (*p*). 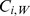
, 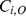 and 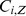 are the clustering coefficient indices proposed by [25], [31] and [9], respectively. 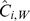, 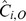 and 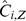 are the corresponding indices generalized to the signed case. **A** Scaled model of the simulated networks. Green lines represent positive edges and red lines represent negative edges. The network in figure includes only 10 nodes, but in the actual simulation we considered larger networks (100 nodes). Moreover, only the edges that were intentionally manipulated are represented. **B** Correlations between the corresponding signed and unsigned indices of the clustering coefficient in the noise-present condition. **C** Correlations between the corresponding signed and unsigned indices of the clustering coefficient in the noise-absent condition. **D** Correlations between different clustering coefficient indices, both signed and unsigned, in the noise-present condition. **E** Correlations between different clustering coefficient indices, both signed and unsigned, in the noise-absent condition.

We generated a simple correlation network in which we regulated the proportion of negative triangles. To create the network, we first generated a matrix of *N* = 100 random variables (1000 observations) from a standard normal distribution. For each variable *i*, we imposed a positive correlation with variable *i*+1, the *N*th variable being correlated to the first variable (correlations were imposed by multiplying a pair of variables by a random variable from a standard normal distribution). We additionally imposed a positive correlation between each variable *i* and the variable *i*+2, the variable *N*−1 being correlated with the first one and variable *N* being correlated with the second one. The matrix 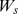 was defined as the correlation matrix, with the diagonal elements set to zero, the nodes therefore represented the variables and the edge weights represented their correlations. This network could be straightforwardly represented using a circular layout (Figure 2A), in which each node *i* was connected to nodes in positions *i*-2, *i*−1, *i*+1 and *i*+2. In this initial network, each node *i* participated in three triangles whose edges were intentionally controlled (we call them *main triangles*) and that had all positive signs. For each node *i*, the three main triangles had vertices i-2, i−1, i; i−1, i, i+1; and i, i+1, i+2. We considered any node *i* as the reference point of the main triangle of vertices *i*−1, i and *i*+1 and used node *i* to define univocally one indirect path (*i*−1, *i*, *i*+1) and one direct edge (*i*−1, *i*+1) for the triangle. We stress that, because of the particular structure of the network, each direct edge corresponded to one and only one main triangle and vice versa, therefore the modification of the direct edge of a main triangle affected only that specific main triangle. We progressively modified the signs of the main triangles, one at a time in random order, by reversing the signs of only the direct edges of the triangles. The proportion of negative triangles was varied until all the main triangles had negative signs. The output of the simulation included 101 networks in which the proportion of negative main triangles ranged from 0% to 100% (Figure 2A).

On average, the absolute weight of the manipulated links was.20 (SD = .03). The networks, however, included noise because of correlations that were not intentionally controlled, which had an average weight of.03 (SD = .02) in absolute value and which were equally distributed among positive and negative edges. Therefore, each node participated additionally in 
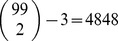
 triangles whose signs were not manipulated but whose weights were small (we call them *random triangles*). In the *noise-present* condition, we computed all of the statistics without removing the random triangles from the network, whereas in the *noise-absent* condition, all edges lower than.1 were set to zero before computing all of the indices of the clustering coefficient, therefore removing most random triangles. The threshold of.1 seems a reasonable choice in the light of the weights distribution. However, we are aware that any fixed threshold has a degree of subjectivity and that other choices could be valid as well. Therefore, we repeated the analyses using a less subjective method by fixing to zero all edges that were not intentionally controlled irrespective of their weight. The pattern of results was substantially similar (see Figure S1), however this procedure did not allow the computation of correlations involving 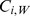 because the index does not vary across nodes. The simulation was repeated 1000 times.

### Results

We computed 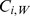, 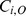 and 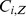, as well as the corresponding signed indices, , 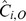 and 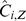, respectively, for each node in each network. In the *noise-present* condition, for the computation of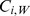 and , an edge was considered present if its weight was higher than.1 in absolute value, absent otherwise. For the computation of the denominator of 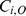 and 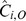, we considered all of the nonzero edges as present in the adjacency matrix. Figure 2B and Figure 2C report the correlation between the corresponding signed and unsigned indices of the clustering coefficient in the noise-present and noise-absent conditions, respectively, as a function of the proportion of negative main triangles induced in the networks. Because the same threshold of.1 was used to manipulate the presence of noise and to compute the unweighted indices of the clustering coefficient, the correlations between 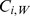 and were identical in the noise-present and the noise-absent conditions, being close to *r* = 1 when only positive main triangles were present, null when both positive and negative main triangles were present in equal proportion, and close to *r* = −1 when only negative main triangles were induced. In the noise-present condition, this pattern was similar, albeit less accentuated for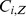 and 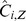. No systematic variation in correlations was present for 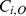 and 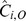 as a function of the proportion of negative main triangles because of the exponents in the numerators of Formulas (3) and (7), which make 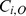 and 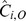 relatively more sensitive than 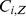 and 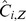 to triangles that are small in absolute weight. Conversely, in the noise-absent condition (Figure 2B), the pattern of correlations was substantially identical for the three indices.

Figures 2D reports the correlation among the different indices in the noise-present condition. The correlation between the unsigned indices was close to zero or negative. In particular, the correlation between 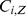 and 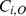 was negative and ranged between r = -.40 and r = -.29. A negative correlation may appear surprising between indices that are meant to assess a similar property, but it can be explained by the different effect that many random triangles have on the two indices, despite their small weight. A positive variation in the absolute weight of the random triangles incident to a node *i* appreciably increases 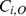 because of the exponent in the numerator of (3) that magnifies the small triangles, but it decreases 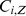 because its effect is stronger in increasing the denominator of (4) than the numerator. Conversely, the correlations between the two signed measures 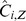 and 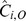 were all positive and ranged between r = .44 and r = .70. The correlation between the signed indices was high and positive, with the exception of the correlation between 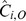 and , which was close to zero. This is because whereas was computed only considering triangles with weights higher than.1, 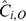 was also affected by triangles with smaller weights.

Figure 2E reports the correlation among the different indices in the noise-absent condition. Removing the noise from the network increased the correlations both between the signed and between the unsigned indices of the clustering coefficient. The correlation between the signed indices was always higher than or equal to the correlation between the corresponding unsigned indices. The reversed “U” shape of the pattern of correlations between the signed indices was attributable to the restriction of range when almost only positive or almost only negative main triangles were present.

### Discussion

To test Hypothesis 1, we inspected the correlations between the corresponding signed and unsigned indices of the clustering coefficient. As expected, the correlations between the clustering coefficient indices varied according to the proportion of negative main triangles. Even a small proportion of negative triangles in a network can make a substantial difference between the indices of a clustering coefficient computed considering or disregarding the edge signs. Consider, for instance, that when the proportion of negative main triangles increased from 0% to 25%, the correlation between the corresponding signed and unsigned indices decreased from 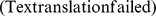 to 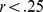 for all of the indices. This trend, however, was apparent for 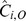 and was more accentuated for 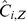 only when the noise was removed from the network because of the influence of many small random triangles on the unsigned weighted indices 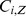 and 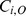.

To better understand the influence of small random triangles on the weighted indices, we inspected the correlation between the weighted indices that considered or disregarded the signs in the noise-present condition (Figure 2D). Whereas the signed indices 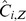 and 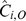 clearly converged, the unsigned indices 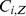 and 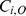 showed a marked divergence. In the computation of the signed indices, positive and negative random triangles tend to cancel each other; conversely, they have an additive effect on the unsigned indices that can obscure the effect of systematic variation. In the noise absent condition, after removing the random triangles, all the indices showed a much stronger convergence (Figure 2E). In conclusion, Hypothesis 2 was also confirmed: the signed indices of the clustering coefficient were more resistant than were the unsigned indices to the presence of random edges.

## Study 2: Clustering Coefficient on Personality Psychology Data

The simulation showed the behavior of the unsigned and the signed indices in a simplified and idealized condition. We tested the behavior of the indices on a real dataset of personality data in which the correlation coefficients could not be divided *a priori* into random and systematic edges.

### Method

There-hundred-fifty-five participants (275 female and 76 male, M age = 23.4, SD = 6.4, plus four participants who did not indicate gender and age) were administered the HEXACO-60 [40], a short 60-item inventory that assesses six major dimensions of personality: honesty-humility, emotionality, extraversion, agreeableness vs. anger, conscientiousness and openness to experience [41]. Moreover, for each dimension there are four facet scores, lower-order traits that are subsumed by the major dimensions: facet scores can be computed as the average of two or three items, depending on the facet [40].

For the item labeling, we followed this convention both in the text and in the figures. We used a letter to indicate the personality dimension that the item measures: H indicates honesty-humility, E indicates emotionality, X indicates extraversion, A indicates agreeableness vs. anger, C indicates conscientiousness and O indicates openness to experience. The items are then numbered in order of administration, the same as was reported by Ashton and Lee [40], in which the complete item content is available.

Twenty-nine items of the HEXACO-60 assessed the negative poles of the traits and were therefore reverse-scored. Reverse-scoring is a typical procedure in scoring questionnaire items that consists in subtracting the item’s score, in this case expressed on a scale from 1 to 5, from the sum of the maximum possible value plus the minimum possible value (in this case, 6). The reverse score expresses the item score as if it assessed the positive pole of the trait.

Ethics statement: The study was approved by the Ethics Committee of the University of Milan-Bicocca. Informed written consent was obtained before testing from all participants involved in the study.

### Results

**Figure 3:**
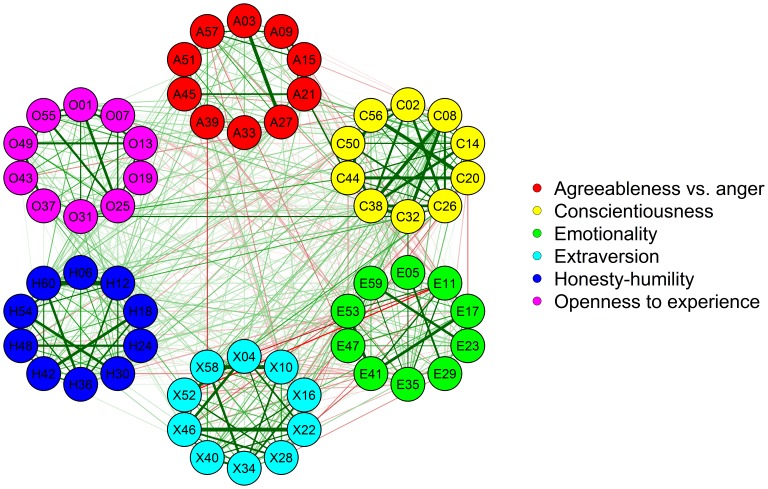
Graphical representation of the network of the HEXACO-60. Items are grouped by the personality factor that they assess. Green lines represent positive correlations, and red lines represent negative correlations.

The 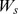 matrix was defined as the correlation matrix between the HEXACO-60 items, and the diagonal elements were set to zero. The resulting network is represented in Figure 3 using the R package *qgraph* [35]. The number of positive edges was 1206, and the number of negative edges was 564. The number of positive triangles was 20600, and the number of negative triangles was 13620. These numbers show that negative triangles are substantially present in empirical data that can be considered typical in personality psychology. However, positive triangles were on average higher in weight than were negative triangles. We defined the weight of a triangle as the product of its edge weights, in absolute value. The average weight of a positive triangle was higher (M = .0018, SD = .0047) than was the average weight of negative triangles (M = .0005, SD = .0007), and this difference was largely significant, as emerged from an independent samples t-test, t(34218) = 32.97, p<10^−15^.

The indices of the weighted clustering coefficient 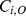 and 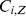, 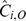 and 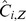 were computed for each node (the values are reported in Table S1). We inspected the correlation among all the unsigned and signed measures of the clustering coefficient (Table 1). The correlations among the corresponding signed and unsigned indices were substantial both between 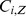 and,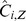 and between 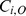 and 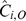. As expected, the signed indices 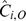 and 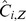 showed a much stronger agreement than the unsigned indices 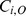 and 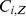, for which the correlation did not reach statistical significance.

**Table 1:** Correlations between the clustering coefficient indices computed on the HEXACO-60.

	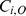	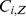	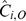	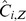
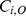	1	.10	.79**	.31*
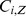	.07	1	.19	.82**
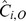	.84**	.08	1	.58**
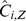	.38**	.80**	.58**	1

*p<.05,

**p<.01. N = 60. Spearman-rank correlations are reported above the diagonal; Pearson’s correlations are reported below the diagonal. 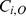 and 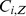 are the clustering coefficient indices proposed in [31] and [9], respectively. 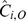 and 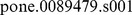 are the corresponding indices generalized to the signed case.

**Figure 4:**
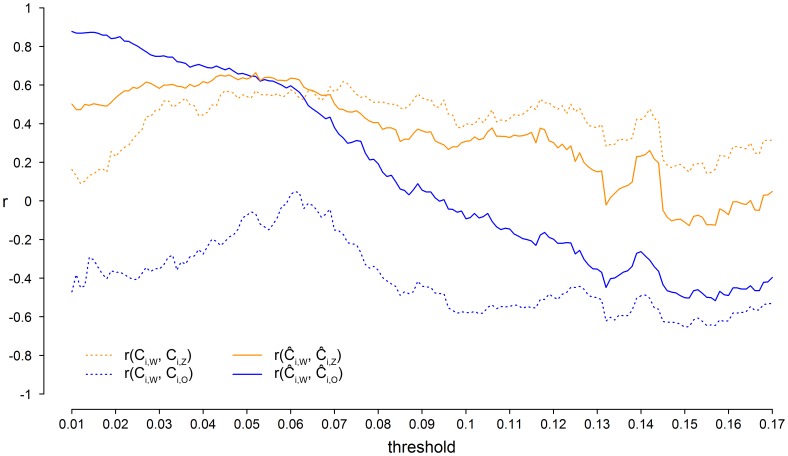
Correlation (r) between the unweighted and the weighted indices of clustering coefficient. 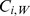
, 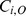 and 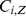 are the clustering coefficient indices proposed by [25], [31] and [9], respectively. 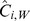, 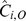 and 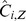 are the corresponding signed indices. 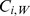 and are unweighted because they do not depend on edge weights and require a binary network, whereas 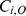, 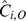, 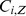 and 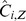 consider edge weights. The correlations are shown as a function of the threshold used for the dichotomization of the network to compute 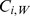 and .

For the computation of 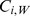 and , a dichotomization was necessary; however, in contrast with the simulation study, it was not possible to select a value that would easily divide the edges into random and systematic. Therefore, we chose to examine the results as a function of different thresholds. Figure 4 shows the correlations between the unweighted indices 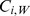 and and the weighted indices 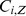, 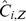, 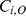 and 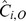 when the threshold varied between a minimum value of.01 and a maximum of.17. Using higher thresholds would not have guaranteed the presence of two neighbors for each node, which is essential to computing the clustering coefficient for every node. Figure 4 shows that the most substantial correlations between the signed indices were reached for low thresholds, but important variations in the agreement of both signed and unsigned indices arose as a function of the threshold.

**Figure 5:**
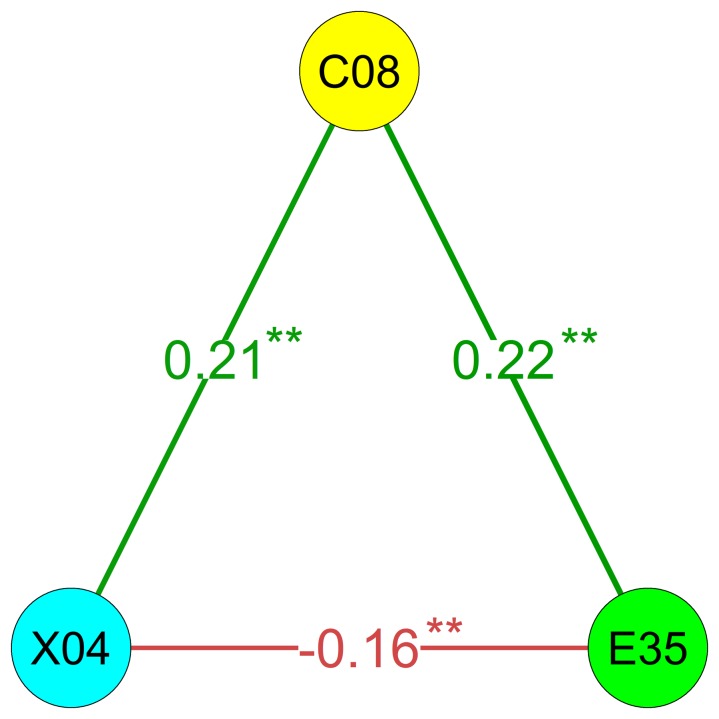
An example of a triangle that emerged from real data. **p<.01. Edge weights are defined as the Pearson’s correlation coefficients among the three items. The letters indicate the personality dimension assessed by the item, C = conscientiousness, E = emotionality and X = extraversion, and the numbers indicate their order of administration on the questionnaire (see [40]).

One reason it is important to consider signed indices is in their potential implications in terms of understanding and interpreting network relations. To provide a psychologically meaningful example, we present a triangle that emerged from the data that can provide some insight on possible interpretative differences between considering and disregarding triangle signs. The triangle (Figure 5) contains nodes C08 (“I often push myself very hard when trying to achieve a goal”), E35 (“I worry a lot less than most people do”; this item is reverse-scored, indicating greater worries) and X04 (“I feel reasonably satisfied with myself overall”). The triangle is discussed from the perspective of node C08 as the focal node (one could equally interpret the direct and the indirect paths using another node as the focal one). The indirect path (E35, C08, X04) suggests that anxiety (E35) is positively related to diligence (C08), which in turn is positively related to social self-esteem (X04). Therefore, if one attempted to predict the direct path (X04, E35) with no knowledge other than the indirect path, one would hypothesize a positive relationship between social self-esteem and anxiety. However, social self-esteem (X04) and anxiety (E35) are negatively correlated: the direct path and the indirect path are not redundant. If one considers the edge signs, one may hypothesize that nodes E35 and X04 are negatively connected *despite* the effect of diligence (C08) but not that they are connected because of it. The same pattern is also present at the level of facets (for facets in the HEXACO, see [40], [41]), with anxiety being positively related to diligence (r = .26, p<.01), diligence positively related to social self-esteem (r = .18, p<.01) and social self-esteem negatively related to anxiety, r = -.34 (p<.01). One could speculate that this triangle reflects a negative feedback loop, as described by Cramer and colleagues [13]. A reasonable level of anxiety can help in focusing one’s goals (e.g., [42]), and pursuing goals may lead to higher self-esteem (e.g., [43], [44]), which in turn reduces anxiety and reinstates the equilibrium. Negative feedback loops are essential in maintaining homeostasis, yet this relationship pattern would have been lost or misinterpreted by disregarding the edge signs when computing the clustering coefficient in correlation networks. In short, disregarding signs can entail the loss or misinterpretation of important information.

### Discussion

This analysis showed how the indices of the clustering coefficient performed when applied to real data from a personality network. Among the weighted indices, the signed indices 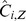 and 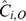 converged with each other whereas the unsigned indices 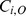 and 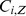 did not show a significant convergence. Hypothesis 2 therefore was also confirmed with real data: a higher convergence was reached when negative triangles were considered with negative signs.

With the real data, it was not possible to find a convincing binary division between systematic and random edges. For the computation of the unweighted indices 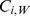 and , we examined different possible thresholds: the indices, and therefore their convergence with the other clustering indices, were noticeably dependent on the selection of the threshold parameter.

## Conclusions

We presented three modified indices of clustering coefficient especially conceived for correlation networks, that account for negative interactions. The new measures have both theoretical and practical advantages: they distinguish positive from negative triangles, which have a different meaning in correlation networks and in psychological data in particular. Moreover, they are more resistant than are the unsigned measures to random sample variation in correlation matrices. The first measure that we introduced, , does not take weights into account and is particularly indicated in the analysis of signed unweighted networks or for those situations in which it is sensible to divide the edges of a weighted network into systematic and random to obtain a binary network. The other two measures, 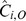 and 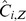, take both weights and signs into account and are particularly useful for the analysis of correlation networks based on real data, in which a clear division between systematic and random edges cannot be performed without substantially affecting the results.

In the psychological data that we considered, positive triangles showed on average higher weights than did negative triangles, causing higher correlations between the signed and the unsigned measures of the clustering coefficient. Personality questionnaires are typically assembled relying on techniques based on the concept of simple factor structure (e.g., [45], [46]): a factor analysis or a principal component analysis is performed, the initial factorial structure is rotated to achieve the simplest possible factor structure given the data, and those items are finally selected that show high primary loadings and low secondary loadings (i.e., the highest item-factor correlation should be much stronger than the second highest) [40], [47]. The strongest correlations in the matrix are therefore among items belonging to the same factor, which form only positive triangles with each other: this is likely to determine the much stronger weight of positive triangles in these networks. Personality questionnaires that have been assembled using different criteria (e.g., [48]–[51]) are expected to produce stronger negative triangles and should be targeted by future research.

The application of network analysis to personality psychology is recent, but it is stimulating important insights in the field that would not have been possible without it [14]. The use of the network approach as a tool for analyzing personality data elicits further substantive considerations on how the network concepts are operationalized and what inferences they allow one to draw [52]. The aim of this contribution is to tailor a number of tools by bearing in mind the specific issues and data that are typical of research in this field with the aim of further extending the use of network analysis as well as of generating novel insights. Although the signed generalizations of the clustering coefficient have originated from this perspective, their potential usefulness and applicability go beyond the realm of personality psychology. In brief, whenever negative triangles can be expected to be present in a network, such as in social networks [21] or biological networks [32], using indices based on signed correlation networks can be quite valuable. They can be particularly useful when there is some level of random noise in the correlation matrix because we have shown that they are much more resistant to such noise than are equivalent unsigned indices. We suspect that these conditions can be present in several other network analysis application contexts and therefore that these proposed indices can have a wide range of applicability for other domains and topics.

## Supporting Information

Figure S1
**Correlation (** ***r*** **) between clustering indices in the alternative noise-absent condition.** The correlations are represented as a function of the proportion of negative triangles (*p*). The noise-absent condition was obtained by excluding all of the edges that were not intentionally controlled in the network. This manipulation of noise is an alternative to the exclusion of the edges of weight lower than a threshold, which is presented in Figures 2C and 2E. Correlations involving 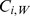 are not represented because this index does not vary across nodes in the alternative noise-absent condition.(TIF)

Table S1 **Descriptive statistics and clustering coefficient by item.**
(DOC)
