# Supplementary figures and images for: Generalization of Clustering Coefficients to Signed Correlation Networks
Source: PLoS One. 2014 Feb 21;9(2):e88669. doi: 10.1371/journal.pone.0088669 (PMC3931641; doi:10.1371/journal.pone.0088669)

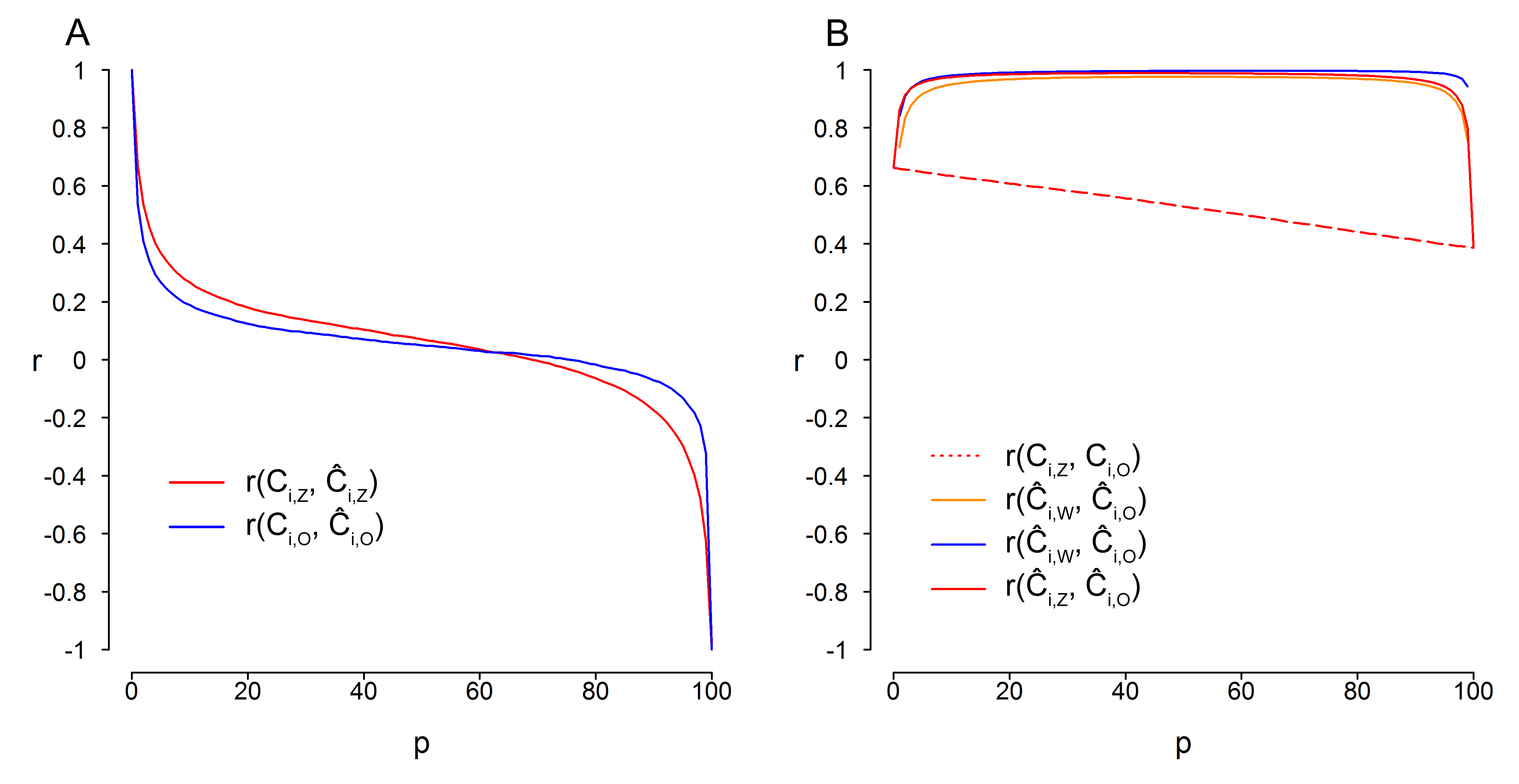

Supplement: Figure S1 — Correlation ( r ) between clustering indices in the alternative noise-absent condition. The correlations are represented as a function of the proportion of negative triangles (p). The noise-absent condition was obtained by excluding all of the edges that were not intentionally controlled in the network. This manipulation of noise is an alternative to the exclusion of the edges of weight lower than a threshold, which is presented in Figures 2C and 2E. Correlations involving are not represented because this index does not vary across nodes in the alternative noise-absent condition. (TIF) [file pone.0088669.s001.tif]
