# Supplementary material for: Generalization of Clustering Coefficients to Signed Correlation Networks
Source: PLoS One. 2014 Feb 21;9(2):e88669. doi: 10.1371/journal.pone.0088669 (PMC3931641; doi:10.1371/journal.pone.0088669)
Supplement: Table S1 — Descriptive statistics and clustering coefficient by item. (DOC) [file pone.0088669.s002.doc]

Supporting Table S1. Descriptive statistics and clustering coefficient by item.

| Item | Mean | SD | $C_{i,O}$ | $C_{i,Z}$ | $\hat{C}_{i,O}$ | $\hat{C}_{i,Z}$ |
| --- | --- | --- | --- | --- | --- | --- |
| A03 | 2,94 | 1,16 | 0,071 | 0,112 | 0,025 | 0,075 |
| A09 | 3,12 | 1,25 | 0,072 | 0,110 | 0,024 | 0,071 |
| A15 | 2,38 | 1,11 | 0,069 | 0,111 | 0,018 | 0,067 |
| A21 | 3,85 | 1,09 | 0,094 | 0,107 | 0,046 | 0,079 |
| A27 | 2,59 | 1,02 | 0,065 | 0,115 | 0,023 | 0,085 |
| A33 | 3,00 | 1,02 | 0,058 | 0,107 | 0,010 | 0,054 |
| A39 | 3,01 | 1,02 | 0,062 | 0,107 | 0,014 | 0,060 |
| A45 | 3,14 | 1,07 | 0,072 | 0,105 | 0,018 | 0,055 |
| A51 | 2,70 | 1,02 | 0,066 | 0,112 | 0,019 | 0,067 |
| A57 | 2,14 | 0,94 | 0,087 | 0,106 | 0,026 | 0,065 |
| C02 | 3,49 | 1,16 | 0,069 | 0,146 | 0,015 | 0,104 |
| C08 | 4,24 | 0,76 | 0,094 | 0,122 | 0,041 | 0,090 |
| C14 | 3,92 | 0,95 | 0,082 | 0,133 | 0,033 | 0,100 |
| C20 | 3,22 | 1,11 | 0,088 | 0,118 | 0,031 | 0,077 |
| C26 | 3,43 | 1,11 | 0,077 | 0,142 | 0,021 | 0,103 |
| C32 | 4,00 | 0,96 | 0,104 | 0,120 | 0,052 | 0,095 |
| C38 | 3,93 | 0,90 | 0,091 | 0,135 | 0,037 | 0,105 |
| C44 | 3,15 | 1,15 | 0,093 | 0,121 | 0,043 | 0,093 |
| C50 | 3,03 | 1,18 | 0,069 | 0,136 | 0,013 | 0,083 |
| C56 | 3,25 | 1,08 | 0,079 | 0,134 | 0,024 | 0,095 |
| E05 | 2,42 | 1,19 | 0,073 | 0,108 | 0,022 | 0,061 |
| E11 | 3,67 | 1,13 | 0,082 | 0,114 | 0,023 | 0,070 |
| E17 | 3,82 | 1,11 | 0,083 | 0,112 | 0,026 | 0,068 |
| E23 | 3,19 | 1,19 | 0,071 | 0,115 | 0,025 | 0,076 |
| E29 | 3,26 | 1,18 | 0,071 | 0,109 | 0,022 | 0,064 |
| E35 | 3,81 | 0,98 | 0,084 | 0,117 | 0,027 | 0,073 |
| E41 | 2,97 | 0,99 | 0,073 | 0,125 | 0,024 | 0,085 |
| E47 | 3,91 | 0,91 | 0,075 | 0,116 | 0,022 | 0,070 |
| E53 | 2,98 | 1,17 | 0,083 | 0,115 | 0,028 | 0,078 |
| E59 | 4,10 | 1,06 | 0,080 | 0,114 | 0,032 | 0,078 |
| H06 | 3,59 | 1,17 | 0,066 | 0,129 | 0,022 | 0,092 |
| H12 | 3,56 | 1,33 | 0,094 | 0,120 | 0,044 | 0,095 |
| H18 | 2,99 | 0,98 | 0,079 | 0,114 | 0,034 | 0,088 |
| H24 | 3,79 | 1,09 | 0,070 | 0,118 | 0,021 | 0,078 |
| H30 | 3,99 | 0,96 | 0,090 | 0,116 | 0,043 | 0,089 |
| H36 | 3,53 | 1,26 | 0,079 | 0,127 | 0,032 | 0,103 |
| H42 | 3,10 | 1,18 | 0,075 | 0,124 | 0,037 | 0,100 |
| H48 | 3,28 | 1,13 | 0,064 | 0,124 | 0,019 | 0,084 |
| H54 | 3,75 | 1,13 | 0,068 | 0,130 | 0,031 | 0,102 |
| H60 | 3,83 | 1,34 | 0,092 | 0,118 | 0,044 | 0,097 |
| O01 | 4,03 | 0,99 | 0,074 | 0,128 | 0,040 | 0,112 |
| O07 | 3,15 | 1,17 | 0,065 | 0,114 | 0,020 | 0,076 |
| O13 | 4,35 | 0,93 | 0,068 | 0,113 | 0,028 | 0,081 |
| O19 | 3,29 | 1,03 | 0,055 | 0,105 | 0,010 | 0,039 |
| O25 | 3,09 | 1,32 | 0,081 | 0,116 | 0,037 | 0,091 |
| O31 | 3,70 | 1,08 | 0,093 | 0,113 | 0,052 | 0,090 |
| O37 | 3,86 | 0,90 | 0,074 | 0,111 | 0,021 | 0,065 |
| O43 | 3,78 | 0,86 | 0,070 | 0,106 | 0,021 | 0,061 |
| O49 | 3,51 | 1,11 | 0,072 | 0,118 | 0,028 | 0,086 |
| O55 | 3,57 | 1,21 | 0,064 | 0,124 | 0,028 | 0,099 |
| X04 | 3,63 | 0,82 | 0,086 | 0,122 | 0,036 | 0,090 |
| X10 | 3,72 | 1,13 | 0,070 | 0,130 | 0,019 | 0,088 |
| X16 | 3,74 | 1,13 | 0,067 | 0,129 | 0,019 | 0,084 |
| X22 | 3,63 | 0,93 | 0,090 | 0,116 | 0,030 | 0,077 |
| X28 | 3,29 | 0,95 | 0,072 | 0,128 | 0,026 | 0,097 |
| X34 | 3,28 | 1,05 | 0,085 | 0,121 | 0,038 | 0,093 |
| X40 | 3,56 | 1,02 | 0,083 | 0,122 | 0,031 | 0,085 |
| X46 | 3,27 | 1,04 | 0,084 | 0,129 | 0,040 | 0,105 |
| X52 | 3,35 | 1,16 | 0,073 | 0,129 | 0,024 | 0,090 |
| X58 | 2,86 | 1,13 | 0,078 | 0,121 | 0,022 | 0,080 |
